# The exploitation of nutrient metals by bacteria for survival and infection in the gut

**DOI:** 10.1371/journal.ppat.1013580

**Published:** 2025-10-30

**Authors:** Summer D. Bushman, Eric P. Skaar

**Affiliations:** Department of Pathology, Microbiology, and Immunology, Vanderbilt Institute for Infection, Immunology, and Inflammation, Vanderbilt University Medical Center, Nashville, Tennessee, United States of America; Carnegie Mellon University, UNITED STATES OF AMERICA

## Abstract

Trace transition metals are required for cellular life processes, such as respiration, metabolism, and DNA replication. At high levels, nutrient metals can be toxic due to oxidative stress and mismetallation of critical metalloenzymes. All organisms tightly regulate intracellular trace metal levels to ensure sufficiency for essential processes while avoiding excess. Microbes including bacteria, viruses, fungi, and archaea colonize hosts forming the microbiota, which in vertebrates is most abundant in the gastrointestinal tract. Invading pathogens compete for metals not only with the host but also with the resident gut microbiota, which provides colonization resistance. To prevent severe infection by enteric pathogens, vertebrate hosts leverage the narrow viable range of trace metal concentrations via both metal sequestration and intoxication in a process called nutritional immunity. In response, microbes have evolved trace metal uptake and export mechanisms to maintain homeostatic levels. In this review, we discuss how the trace transition metals iron, zinc, manganese, and copper influence the composition of the gut microbiota and its subsequent ability to compete with enteric pathogens. We explore the specialized mechanisms that pathogens employ to successfully acquire nutrient metals during infection in the gut and describe how these systems could be exploited for therapeutic development. Finally, we report the powerful mechanisms utilized by the microbiota to compete with enteric pathogens for metals and how they can inspire potential antipathogenic tools.

## Introduction

Trace transition metals, such as iron (Fe), zinc (Zn), manganese (Mn), and copper (Cu) are critical for all domains of life. Metals are crucial to stabilize protein structures, promote catalysis as enzyme cofactors, and facilitate cell signaling and metabolism [1,2]. Simultaneously, excess metals are cytotoxic through mismetallation of essential proteins and production of chemical species that cause oxidative damage [3]. The inability to synthesize trace metals and the critical role they play in various cellular processes emphasizes the need for careful maintenance and regulation. Both vertebrate and microbial cells have evolved mechanisms to sense, import, and export nutrient metals to maintain homeostasis.

The trillions of bacteria that colonize the vertebrate gastrointestinal (GI) tract are termed the gut microbiota and consist of over 1,000 different species, with Bacillota (previously Firmicutes) and Bacteroidota as the most abundant phyla [4,5]. The gut microbiota not only aids in host digestion and metabolism but also affects intestinal permeability and contributes to the vertebrate immune, endocrine, reproductive, and nervous systems [6–10]. In particular, the gut microbiota provides the host with protection from invading and opportunistic pathogens through colonization resistance [11]. Although the full range of colonization resistance mechanisms remains unclear, they include niche occupation, nutrient competition, secretion of antimicrobial molecules, and maintenance of the intestinal barrier [12]. When gut microbiota diversity is reduced or imbalanced the gut enters a state called dysbiosis. Under these conditions, a member of the gut microbiota that is typically a commensal may become an opportunistic pathogen and cause disease [13]. Antibiotic use, improper diet, or exposure to environmental toxins may lead to dysbiosis. Nutrient trace transition metal availability in the early life of vertebrates modulates microbiota composition and community succession, with host nutrient metal deficiencies associated with dysbiosis [14–16]. A perturbed gut microbiota reduces colonization resistance, which is further associated with infectious and chronic diseases [11,17,18].

Despite the presence of a healthy gut microbiota pathogens can still cause disease. Following infection, inflammation leads to the production and recruitment of host immune proteins that bind metals to restrict microbial growth. This process minimizes severe infection by creating a metal-deplete environment and is termed nutritional immunity [19]. Enteric pathogens, opportunistic pathogens, and gut commensals have evolved metal uptake and storage mechanisms to effectively compete and persist in the metal-depleted gut [20]. The battle for trace transition metals at the microbe host-interface and the importance of nutrient metals for the gut microbiota have been well demonstrated [19,21]. This review will focus on the mechanisms of microbe-microbe competition over different nutrient metals among the vertebrate gut microbiota, opportunistic pathogens, and enteric pathogens.

## Iron (Fe)

Fe plays a role in critical cellular and tissue processes including redox reactions, respiration, and metabolism [22]. However, excess Fe in the presence of oxygen can participate in Fenton reactions to generate free radicals, therefore the amount of intracellular Fe is tightly regulated in both host and bacterial cells [23,24]. Fe is often complexed with host compounds, like heme and ferritin, to prevent oxidative stress and damage [25]. Aerobically, the insoluble ferric form (Fe^3+^) dominates, but under hypoxic and anaerobic conditions like the GI lumen, the soluble ferrous form (Fe^2+^) is more prevalent [26]. Regardless of oxidation state, gut bacteria must acquire sufficient Fe from the GI environment, which the host obtains from its diet. Bacteria have mechanisms to remove Fe from vertebrate hemoproteins, such as hemoglobin, and Fe-binding serum proteins, such as transferrin. Bacteria also synthesize low-molecular weight secreted compounds with high Fe-binding affinities, called siderophores, that capture extracellular Fe. The Fe-siderophore complex is then internalized through specific receptors [27]. Across bacterial species, many genes that encode Fe import mechanisms are regulated by the *f*erric *u*ptake *r*egulator (Fur). Canonically, Fur represses expression of genes encoding for Fe or heme acquisition machineries under Fe-sufficient conditions, and transcription is derepressed in low Fe conditions [28]. Fur enables the microbiota to persist in the vertebrate gut and contributes to microbial pathogenesis by facilitating the bacterial response to Fe-limited conditions created by nutritional immunity [29]. Once Fe is imported into the bacterial cell through these acquisition systems, it may be stored in proteins such as bacterial ferritin, bacterioferritin, or ferrosomes to prevent the formation of reactive oxygen species (ROS) from hydroxyl radicals [30–32]. There is a bidirectional relationship where vertebrate Fe levels can influence the gut microbiota composition, and the gut microbiota modulates the amount of bioavailable Fe and host Fe levels in the GI tract [33].

### Fe and the gut microbiota

Approximately 10^5^–10^6^ ions of Fe are required per a typical bacterial cell, so preserving stable Fe levels in the GI tract is important [34]. In fact, the fecal microbiota composition and metabolome of mice fed diets with varying levels of Fe for 7 weeks remained distinct after all groups were switched to a standardized diet for 7 weeks [35]. This finding indicates that dietary Fe deficiencies or overloads have long-lasting impacts on the gut microbiota. Further, dysfunctional Fe regulation in the vertebrate host is associated with dysbiosis, which in turn is associated with metabolic syndromes including obesity and type 2 diabetes [36]. Excess iron in the serum is associated with increased insulin resistance, obesity-associated metabolic signatures, and a disrupted gut microbiota, creating a feedback loop between the gut microbiota and host health [37]. The gut microbiota must therefore have strategies to be resilient to variations in dietary iron, including gut commensals such as *Bifidobacteria* spp*.*, which utilize siderophores to maintain their Fe requirements [38,39].

Common short chain fatty acids (SCFA), butyrate, acetate, and propionate, are major metabolites produced by the gut microbiota that reduce inflammation [40]. Fe levels in the GI tract can modulate the gut microbiota composition, which can alter SCFA concentrations. Fe deficiency lowered the abundance of the SCFA producer, *Roseburia intestinalis,* both *in vitro* and during colonization of Fischer rats, resulting in lower intestinal butyrate [41]. Oral Fe supplementation consequently increased gut butyrate levels [41,42]. These findings contrast with another study that reported Fe-deficient rats had increased SCFA levels in their colons compared with Fe sufficient rats [43]. There were strain- and sex-dependent differences between these studies which may impact the conclusions about the impact of Fe on SCFA levels. Heme supplementation in mice also depleted butyrate-producing Clostridial species, shifting the microbiota toward dysbiosis [44]. Similarly, high dietary hemin, an oxidized form of heme, resulted in a disruption of murine small intestine bacterial flora including higher relative abundance of *Turicibacter,* which is associated with inflammatory cytokines, and toxicity toward beneficial genera such as *Bifidobacterium* and *Lactobacillus*. Furthermore, a high hemin diet induced colonic barrier disruption and duodenal injury, which may render the host more susceptible to enteric infection [45]. However, if members of the gut microbiota are already subjected to iron deprivation, then moderate heme supplementation can prevent taxon depletion and stabilize the community structure of the microbiota [46]. Given the essentiality and toxicity of Fe, the most diverse gut microbiota with sufficient SCFA production thrives with a moderate amount of Fe.

Excess Fe in rats decreases intestinal integrity, increases oxidative stress, and alters microbiota composition [47]. Specifically, excess Fe reduces the abundance of Bacillota and *Bacteroides* spp. [48]. Decreases in Bacillota are associated with increased *Clostridioides difficile* infection (CDI) and nosocomial diarrhea from other enteric pathogens [49]. Moreover, oral administration of excess Fe after antibiotic exposure delays the recovery of the gut microbiota [50]. These findings suggest there is an upper limit to beneficial Fe concentrations, but the exact value may vary by bacterial species.

### Fe in enteric infections

**Fig 1:**
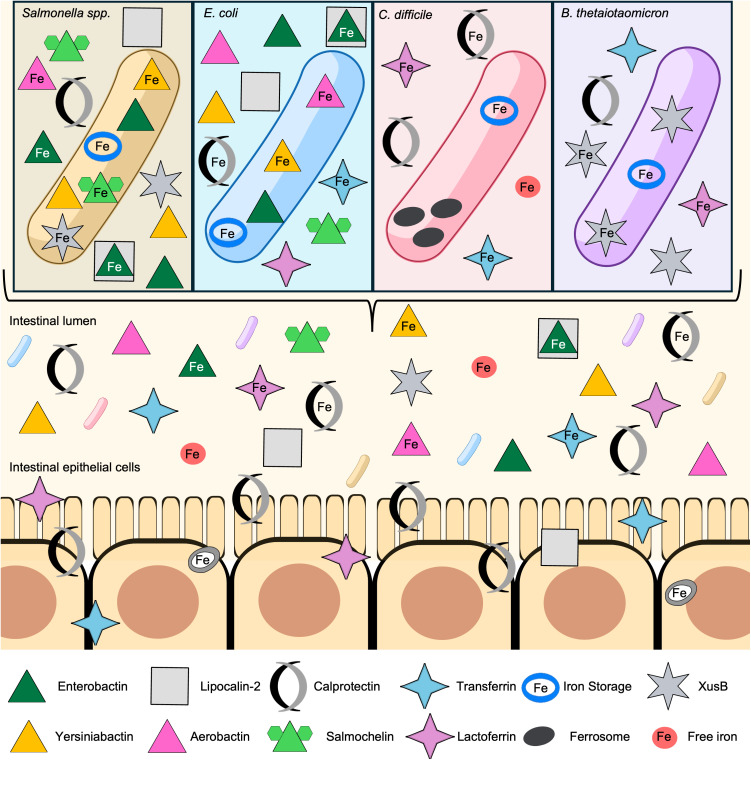
Competition for Fe in the vertebrate gut. Inflammation from infection promotes Fe sequestration by host proteins like calprotectin, transferrin, and lactoferrin. Pathogens such as *Salmonella* as well as commensals including *E. coli* secrete siderophores to capture Fe in the intestinal lumen. Some of these are stealth siderophores like salmochelin, which is unable to be bound by lipocalin-2. The host can store Fe in cage-like iron storage molecules and similarly, some gut pathogens, such as *C. difficile*, store Fe in ferrosomes to survive host-mediated Fe limitation. There are also xenosiderophore transport systems like XusB, which provide a competitive advantage to *B. thetaiotaomicron*.

Vertebrates possess several proteins, such as lactoferrin, transferrin, lipocalin-2, and calprotectin, which participate in nutritional immunity and limit available divalent metals, including Fe [51,52]. The impact of nutritional immunity is demonstrated by the observation that limited Fe lowers the abundance of enteric pathogens and may prevent severe infections [53]. Bacteria often encode siderophores to acquire sufficient Fe, which is essential to survive the low Fe environment imposed by nutritional immunity during enteric infection [24]. Enterobacteriaceae members, including the pathogen *Salmonella enterica*, the opportunistic pathogen *Escherichia coli*, and commensal species, encode the siderophore enterobactin, which has the highest Fe binding affinity of all known siderophores and nutritional immunity proteins [54–56]. A highly conserved mechanism in gram-negative bacteria to import Fe-siderophore complexes are TonB-dependent transporters [57,58]. The inflammation triggered by infection leads to the production of IL-17 and IL-22, which stimulates the vertebrate host to express lactoferrin and lipocalin-2 [59]. Lipocalin-2 binds to enterobactin, decreasing its uptake by *Salmonella* and *E. coli* [60] (Fig 1).

Some bacteria possess stealth siderophores that can evade the vertebrate nutritional immune system, ensuring the bacterium maintains sufficient Fe levels. For example, *Salmonella* evolved a glycosylated enterobactin, named salmochelin, which avoids capture from lipocalin-2 [61,62] (Fig 1). The competition is not limited to vertebrate proteins, as probiotic *E. coli* strain Nissle can outcompete *Salmonella* in a murine infection model using the siderophore aerobactin and the stealth siderophore yersiniabactin [63,64]. However, due to horizontal gene transfer, *Salmonella* serovars that produce yersiniabactin, including serovar Infantis are emerging globally, which poses a challenge for the development of competitive probiotics [65]. *Bacillus anthracis,* a pathogen that can cause gastrointestinal infections, use a similar approach and produces two siderophores to capture Fe [66]. One siderophore, bacillibactin, can be bound by the vertebrate innate immune protein siderocalin, but petrobactin is a stealth siderophore [67]. Petrobactin is required for growth of *B. anthracis* in macrophages and pathogenesis in murine infections [68,69]. Therefore, stealth siderophores are an effective tool employed by pathogens to evade the host immune system and compete for Fe in the limited bioavailable Fe pool.

Production of siderophores and their corresponding uptake systems is energy-intensive, so some bacteria may use hijacked siderophores they did not produce, known as xenosiderophores, as an Fe source [70–73]. An abundant member of the gut microbiota, *Bacteroides thetaiotaomicron*, encodes the *x*enosiderophore *u*tilization *s*ystem (Xus) operon [74]. XusB is a lipoprotein that can be incorporated into outer membrane vesicles and can bind enterobactin loaded with Fe, which is then imported by XusA, a TonB-dependent transporter. The Xus operon enables *B. thetaiotaomicron* persistence under the inflammatory and Fe-limited conditions that are common in enteric infections. However, *Salmonella* can “re-acquire” enterobactin bound to XusB to evade nutritional immunity, illustrating an example of direct competition between the gut microbiota and enteric pathogens for Fe [75] (Fig 1). Another GI pathogen, *Vibrio cholerae*, uses enterobactin as a xenosiderophore and this process leads to increased survival in Fe-limited conditions within mice when co-infected with *E. coli* [71]. In addition to xenosiderophores, many bacteria can directly bind host-derived proteins lactoferrin, transferrin and calprotectin, engaging in Fe piracy from the host [76]. Fe piracy acquisition mechanisms have been described in pathogenic gut bacteria, such as *Campylobacter jejuni*, *Salmonella*, and *E. coli* [76–78]. These findings highlight that enteric and opportunistic pathogens successfully combat nutritional immunity via xenosiderophores and Fe piracy to obtain sufficient Fe in the inflamed gut and cause infection.

Similarly, the enteric pathogen *C. difficile* can grow with xenosiderophores as the sole Fe source even though most strains cannot produce siderophores, suggesting the presence of uptake mechanisms. Consistent with this, *C. difficile* possesses an *A*TP-*b*inding *c*assette (ABC) transporter FhuDBGC that binds and mediates uptake of the siderophore ferrichrome [79]. When *C. difficile* encounters an Fe-deficient environment, its transcriptome is altered to upregulate virulence factors, such as flagellar systems and polyamine biosynthesis and uptake [80]. To cope with Fe limitation, *C. difficile* utilizes membrane-bound Fe storage organelles called ferrosomes. The membrane protein FezA and transporter FezB are required for ferrosome formation and are repressed by Fur [81]. This discovery represents a rare demonstration of a bacterial organelle and the first description of ferrosomes in a gram-positive bacterium and a human pathogen (Fig 1). A mutant inactivated for *fezB* had reduced colonization in a mouse model of infection, indicating that ferrosomes and iron storage in general contribute to the ability of *C. difficile* to colonize a vertebrate host. Simultaneously, human patients with CDI have significantly lower levels of Fe in their stool, suggesting that *C. difficile* induces metal dysregulation in the host, which may alter the composition of the gut microbiota [81].

Appropriate iron storage is important for the virulence of enteric pathogens. *Salmonella* Typhimurium possesses four major iron storage proteins, including *D*NA-binding *p*rotein from *s*tarved cells (Dps) and ferritin-like protein B (FtnB). Mice infected with *S.* Typhimurium with *ftnB* or *dps* inactivated have reduced bacterial burden in the liver and increased survival. These results may be partially explained by the *dps* mutant having increased sensitivity to H_2_O_2_-mediated oxidative stress, which may make this mutant more susceptible to the host immune system [82]. This indicates that these iron storage mechanisms contribute to the colonization and fitness of *Salmonella*. In accordance, *C. jejuni* strains with inactivation of ferritin or *dps* exhibit increased sensitivity to oxidative stress, emphasizing the potential role of iron storage proteins in virulence [83,84]. An *E. coli dps* mutant has lower competitive fitness compared with other *E. coli* strains, which indicates that *dps* could contribute to the ability of *E. coli* to colonize within the GI tract [85]. The ability of bacteria to store iron safely is beneficial because it may offer protection from Fe-mediated oxidative damage. Furthermore, having a reservoir of iron during infection may offer a competitive advantage since inflammation creates iron limitation.

These Fe storage proteins may be antimicrobial targets, but caution must be taken as bacterial ferritin proteins share structural motifs with eukaryotic ferritin, therefore the heme-binding bacterioferritin may be a more promising antimicrobial target [86]. Dps is a highly conserved protein found in across bacteria including all gammaproteobacterial orders [87]. The conservation indicates that a therapeutic targeting these proteins could have nonspecific consequences that impact the gut microbiota. In fact, Dps and Dps-like (DpsL) proteins in the opportunistic pathogen, *Bacteroides fragilis* contribute to its ability to survive in prolonged oxidative stress [88]. Dps, DpsL, and the bacterial ferritin protein FtnA are highly conserved among *Bacteroides* and *Parabacteroides* species, many of which are gut commensals, suggesting a potential pitfall of antimicrobials that target Fe storage proteins [89].

During CDI, *C. difficile* toxins damage colonic epithelial cells, causing the epithelium to release the heme-containing hemoglobin [90]. Heme can be a source of Fe but it can be toxic via membrane disruption and DNA damage [91]. The increase in heme concentrations results in the expression of the *C. difficile* heme-detoxification system HatRT where HatT is a heme exporter that contributes to *C. difficile* pathogenesis in an murine infection model [90]. Interestingly, *C. difficile* also possesses HsmRA which simultaneously senses and detoxifies heme, and HsmA protects against oxidative stress. These functions allow HsmRA to contribute to *C. difficile* colonization and persistence in the murine gut [92]. *C. difficile* induced release of heme also enhances the fitness of the opportunistic pathogen, *Enterococcus faecalis* [93]. In this way, CDI further promotes dysbiosis and susceptibility to enteric infections. In contrast to the detoxification systems of *C. difficile*, *B. anthracis* possesses two hemophores, IsdX1 and IsdX2, which mediate heme acquisition from vertebrate hemoglobin and are necessary for growth of *B. anthracis* in low-iron environments [94].

### Future directions for Fe research

Further research into the relationship between host Fe levels and the gut microbiota should incorporate longitudinal studies to discern the long-term impacts of dietary Fe levels. There is also a need for additional cross-sectional studies that account for diverse gut microbiotas, perhaps by inoculating germ-free mice with human fecal samples. Spatial imaging techniques, such as imaging mass spectrometry may help parse out which gut microbiota members contribute to the pool of Fe available in the GI tract and provide anatomical context to the microbial competition for Fe [95–97]. To elucidate competition for Fe beyond population-based studies of the gut microbiota, specific microbe-microbe interactions should be interrogated using synthetic microbiota communities and gnotobiotic animal models of infection. Microbial interaction studies have the potential to describe additional Fe uptake mechanisms that represent possible therapeutic targets. Further research should investigate if other pathogens or commensals have ferrosomes and identify if and how Fe storage mechanisms contribute to bacterial survival in Fe-depleted conditions.

Notably, the commensal *Lactobacillus plantarum* increases Fe absorption in the intestines of Fe-deficient humans more than oral Fe supplementation alone [98]. The potential to develop a commensal-derived probiotic as a prophylactic or adjunct to treatment warrants future research, as this supplement could modulate host Fe levels and/or prevent enteric infections through Fe restriction. Lastly, future studies should look beyond bacteria, as competition within the gut for Fe occurs not only between the vertebrate host and bacteria, but also among fungi and archaea. For example, *Salmonella* can use fungal xenosiderophores to colonize the gut [99]. These findings highlight the importance to consider all members of the gut microbiome and their crosstalk when determining how to best prevent enteric infection and maintain a healthy gut microbiota.

## Zinc (Zn)

**Fig 2:**
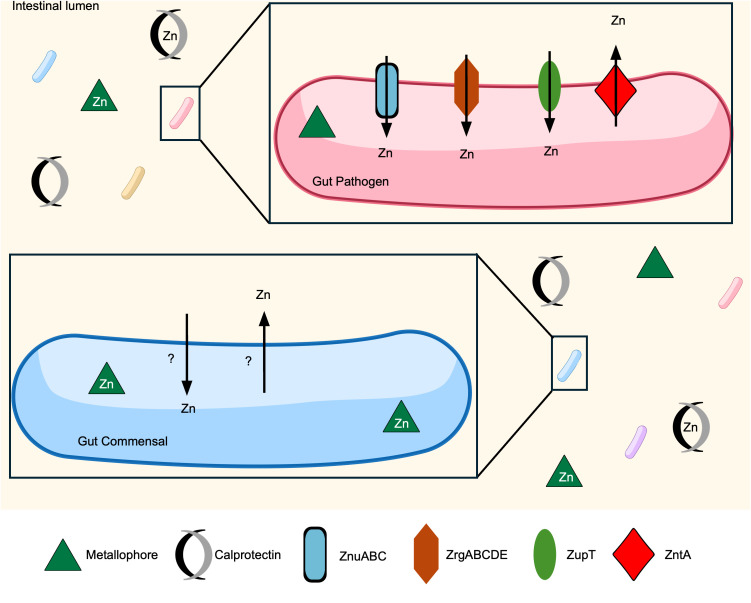
Competition for Zn in the vertebrate gut. Inflammation leads to host-imposed nutritional immunity, which leads to low Zn availability in the GI tract. Enteric pathogens have evolved several Zn uptake mechanisms, such as import from ZnuABC, ZrgABCDE, and ZupT. Some possess the Zn efflux system ZntA to protect against Zn toxicity. Some metallophores, including yersiniabactin, allow native gut microbiota members to persist in times of infection. The exact mechanisms of Zn uptake and export in gut commensals remains to be elucidated.

Zn is essential for all domains of life due to the requirement for many processes including DNA replication and gene expression. Zn provides structural stability for enzymes and can be directly involved in catalysis [100]. Gut microbiota and enteric pathogens must scavenge Zn within the host GI tract to maintain homeostasis [101–103]. While it is critical that bacteria have sufficient levels of Zn, excess Zn can be toxic due to mismetallation of proteins [104]. The vertebrate innate immune system exploits Zn toxicity by accumulating Zn in neutrophils and macrophages, which simultaneously intoxicates engulfed pathogens and restricts extracellular Zn to prevent further proliferation [105]. Zn levels within the bacterial cell must therefore be tightly regulated via Zn uptake and efflux systems [106]. Across different bacterial species, conserved Zn uptake systems are ZnuABC and ZupT, and prevalent exporters are ZntA and ZitB [107] (Fig 2). The exact mechanisms of Zn homeostasis are species-specific, but many of these transport systems are regulated by the Fur-family repressor, *Z*n *u*ptake *r*egulator (Zur) [108]. Both pathogenic and commensal bacteria use these regulatory and transport systems to compete with each other and the vertebrate host to obtain sufficient levels of Zn.

### Zn and the gut microbiota

Organismal Zn levels can be stratified into three groups: Zn-deficient, Zn adequate, and Zn overload, and bacteria in the colon mediate the bioavailability of Zn [109,110]. Since Zn is an essential micronutrient, the finding that both Zn deficiency and Zn overload have profound impacts on the gut microbiota is unsurprising [111]. Zn requirements can double during pregnancy and lactation, making pregnant women at a higher risk for Zn deficiency [112,113]. Zn deficiency in pregnant mice decreases the abundance of bacteria from the phyla Verrucomicrobia and Proteobacteria and increases gut permeability and neuroinflammation [114]. In a non-pregnant vertebrate gut during Zn deficiency, Verrucomicrobia and Clostridiales are depleted while Enterobacteriaceae and Ruminococcaceae increase [115–117]. Enterobacteriaceae is the family that contains *E. coli* and *Salmonella* and is associated with dysbiosis [118]. Additionally, depletion of Verrucomicrobia may be detrimental because certain species within this phylum, such as *Akkermansia muciniphila*, are associated with anti-inflammatory markers [119]. Verrucomicrobia relative abundance is augmented in high Zn diets, indicating that the abundance of this phylum correlates with Zn levels [120]. Further, Zn-deficient children have lower abundance of the probiotic genus *Bifidobacterium* and higher levels of pro-inflammatory metabolites like taurocholate [121]. Interestingly, taurocholate is a known germinant of *C. difficile*, and induces toxin production within *V. cholerae*, potentially rendering those who are Zn-deficient more susceptible to enteric disease [122,123].

Dietary supplementation of Zn oxide (ZnO) in pigs to prevent deficiency, decreased the abundance of *Clostridium* spp. and Enterobacteriaceae. Another study specifically found a reduction in Clostridial Cluster XIa, containing *C. difficile,* after Zn supplementation [124–126]. Both studies found improved intestinal integrity in the groups fed ZnO [124,126]. On the contrary, diets supplemented with Zn chloride increased the abundance of Clostridiaceae, which resulted in improved intestinal integrity and decreased pro-inflammatory cytokines [127]. Importantly, the families Enterobacteriaceae and Clostridiaceae consist of both commensal and pathogenic bacteria. Since these studies did not report species-level resolution, teasing apart the true impact of dietary Zn supplementation on gut microbiota species is challenging. Further, when the antimicrobial peptide bacitracin was supplemented with Zn, 15-day-old broiler chickens had increased gut microbiota diversity [128], but Zn hydroxychloride decreased diversity in broilers [129]. More recently, it was found that different dietary Zn levels did not significantly change the gut microbiota composition of gestating cows [130]. These inconsistencies can be explained by different host organisms and Zn conjugates, which are known to result in distinct biological activity [131,132]. Altogether, these data demonstrate that altered dietary Zn levels may impact the gut microbiota distinctly in different model organisms, underscoring the importance for tight regulation of Zn homeostasis.

While Zn deficiency promotes dysbiosis that may be rescued by ZnO supplementation, Zn overload may also perturb the gut microbiota. Indeed, a human colon simulator exposed to increased levels of ZnO for 7 days exhibited reduced bacterial abundance and diversity in the gut microbiota, with decreases in the commensal SCFA-producing genus *Ruminococcus* [133]. Consistent with this, chronic Zn toxicity in mice resulted in a dysbiotic bloom of Enterobacteriaceae [134]. Taken together, these data indicate that excess Zn can alter gut microbiota diversity, emphasizing the importance of maintaining balanced Zn levels in the host diet. Additionally, while baseline studies on Zn in the gut are informative, more studies should be done in gestating and elderly vertebrates, as these groups are more likely to face Zn deficiency [135,136].

### Zn in enteric infections

In addition to impacting the diversity and abundance of commensals in the gut microbiota, Zn levels play a role in enteric infections. Host Zn deficiency leads to worse infection outcomes due to the requirement of Zn in immune cell function [137]. ZnO is a supplement in the domesticated porcine diet because it improves the gut epithelial barrier and reduces the incidence of diarrhea [109]. However, excess Zn creates a pool of nutrients that allow pathogens to proliferate. Therefore, vertebrates regulate the availability of metals via nutritional immunity through proteins such as lactoferrin and calprotectin [19]. Calprotectin is abundant in neutrophils, comprising 40–50% of total protein content, and has two sites capable of binding Zn [138,139]. Mice lacking calprotectin and human infants with lower levels of fecal calprotectin have a higher abundance of Enterobacteriaceae in their gut [140]. It is important to note that calprotectin binds other metals, including Fe and Mn, so these differences may not be solely due to Zn [138]. This competition between bacteria and vertebrates for Zn and the alteration to the gut microbiota has created evolutionary pressure for bacteria to develop systems to uptake Zn and colonize the gut [141].

The vertebrate immune system relies on Zn for proper function, so Zn deficiency may result in higher susceptibility to enteric infections [142]. Additionally, rats deficient in Zn and challenged with *V. cholerae* have four times greater cholera toxin-induced secretions compared with non-Zn deficient rats [143]. One potential reason why Zn-deficient rodents are so susceptible is because *V. cholerae* has two Zn uptake systems, ZnuABC, and ZrgABCDE. Both are Zur-repressed, allowing the bacteria to quickly respond to low Zn levels and uptake the limited Zn available in the environment. ZnuABC and ZrgABCDE have a role in colonization of infant and adult mouse models, implying that these two Zn uptake systems allow *V. cholerae* to outcompete other gut microbiota members for Zn [144]. Similarly, ZnuABC is essential for *S. enterica* to grow in Zn limited conditions [145]. *S. enterica* also possesses the Zur-regulated protein ZinT which facilitates Zn acquisition by the ZnuABC system, potentially acting as a metallochaperone [146]. *C. jejuni* inactivated for *znuABC* can only infect chickens experiencing dysbiosis, indicating Zn uptake via ZnuABC is essential for *C. jejuni* to overcome colonization resistance [103] (Fig 2).

Enteric pathogens also experience Zn intoxication in macrophages. Bacteria thus possess Zn efflux systems to overcome the stress of Zn overload [105]. The Zn exporter, ZntA, is important for *Vibrio parahaemolyticus* to survive excess Zn and contributes to its virulence in mice [147]. Bacteria without adequate strategies to efflux Zn may succumb to Zn toxicity. Broiler chickens with dietary ZnO supplementation had significantly less bacterial burden of the opportunistic pathogen *Clostridium perfringens* in their GI tracts compared with broilers who did not receive ZnO supplementation, making Zn toxicity a method to limit gut pathogens like *C. perfringens* [148].

*E. coli* can be a beneficial member of the gut microbiota or a pathogen depending on the strain [149]. The probiotic strain *E. coli* Nissle, which possesses the metallophore yersiniabactin, can resist calprotectin-induced Zn sequestration better than the pathogenic bacterium *Salmonella* Typhimurium, partially explaining its beneficial properties [72]. Zn also plays a significant role in the virulence of the pathogenic *E. coli* strains. Piglets receiving dietary ZnO supplementation have less inflammation when challenged with enterotoxigenic *E. coli* [150,151]. Further, dietary Zn supplementation, via ZnO or Zn phosphate, results in less adhesive *E. coli* [152,153]. The production of alpha-hemolysin, an exotoxin produced by some pathogenic *E. coli* strains, is affected by Zn levels. Zn ions prevent alpha-hemolysin-induced barrier dysfunction in the porcine intestinal epithelium by binding directly to the mucosa [154,155]. Piglets fed ZnO have less enterotoxigenic, enteropathogenic, and Shiga toxin-producing *E. coli* pathotypes in their GI tract compared to those fed a control diet [156]. Altogether, these results suggest that Zn deficiency increases susceptibility to enteric infection by *E. coli* and that Zn supplementation is protective. Zn reduces the virulence of *E. coli*, but whether this is because Zn exhibits antimicrobial effects against the virulent strains or because the strains become more virulent when they are starved for Zn remains to be elucidated.

The host diet, including Zn availability, influences CDI, and *C. difficile* is known to compete with the gut microbiota for nutrients [157,158]. About 20% of *C. difficile*-infected patients develop recurrent infections, often due to continued dysbiosis which is impacted by Zn [159]. In a small trial, patients with low Zn levels had a recurrence rate of about 16%, while those with Zn levels in a normal range had a recurrence rate of 6%, indicating that Zn deficiency plays a role in CDI recurrence [160]. Additionally, there are case studies of patients failing to clear recurrent *C. difficile* until their antibiotic treatment is Zn supplemented [161]. Interestingly, patients that develop recurrent CDI have higher levels of the immune metal sequestration proteins calprotectin and lactoferrin in feces from their first episode of CDI compared with those who do not develop recurrent CDI [162]. Calprotectin is a marker for severe CDI and creates a metal-limited environment in the GI tract that minimizes *C. difficile* pathogenesis [163]. In fact, mice unable to produce calprotectin do not survive CDI, so calprotectin is essential for the host to combat CDI [96]. To compete with nutritional immunity, *C. difficile* encodes the Zn importer, ZupT [164]. While Zn limitation reduces CDI severity, excessive dietary Zn increases *C. difficile* toxin production and disease severity in mice [96]. Furthermore, excess Zn alters the gut microbiota composition, facilitating *C. difficile* colonization [141]. Therefore, Zn plays a significant role in CDI and the role of Zn in other enteric infections warrants further research.

### Future directions of Zn research

Dietary Zn supplementation reduces the incidence of diarrhea in weaned livestock and human children, which is likely due to Zn sufficiency leading to proper immune function, intestinal integrity, and a robust gut microbiota that provides colonization resistance [109,165]. Zn can also block basolateral membrane potassium channels, which prevents excess water secretion in the intestines [166]. Some proteins can bind several metals, as is the case for calprotectin, yet Zn is often studied independently of other nutrient metals [138]. One proposed mechanism for Zn protecting against bacterial infections suggests that Zn inhibits pathogenic bacteria from obtaining sufficient Mn. Zn can bind to Mn acquisition proteins and lock them in a closed state that prevents Mn transport [167]. Therefore, multiple metals should be considered in the context of the battle for nutrient metals between host and pathogen due to the possibility of metal crosstalk or synergism.

There is potential that gut microbiota members can be used to diagnose Zn deficiency. Currently, serum is used to diagnose Zn deficiency, but collecting a stool sample that is representative of the gut microbiota could be less stressful than serum, especially for children or animals [168]. *Desulfovibrio* spp*.* and gut commensals from the phylum Melainabacteria are proposed markers of mammalian Zn status. Their abundances correlated with mouse serum Zn levels in both short and long-term dietary interventions [120]. With further validation, diagnostics could be developed using these bacterial biomarkers to identify people who are Zn-deficient. Additionally, gut commensals could be used as a treatment that directly compete with enteric pathogens. Probiotic *E. coli* Nissle is resistant to calprotectin-mediated Zn limitation, but a strain that is able to reduce enteric pathogen burden or virulence has clinical potential [72]. This illustrates the therapeutic potential of gut microbiota members working in synergy with the host immune system to starve enteric pathogens of nutrient metals. Moreover, if formulated as a probiotic given to patients during antibiotic use, this could be a preventative therapeutic. Future research could explore the concept of engineering gut commensals that mitigate dysbiosis and/or enteric infection severity by competing for metals. Overall, receiving adequate levels of Zn through the diet, compared to Zn deficiency or overload, is the best way to maintain a healthy gut microbiota composition and prevent enteric infection.

## Manganese (Mn)

**Fig 3:**
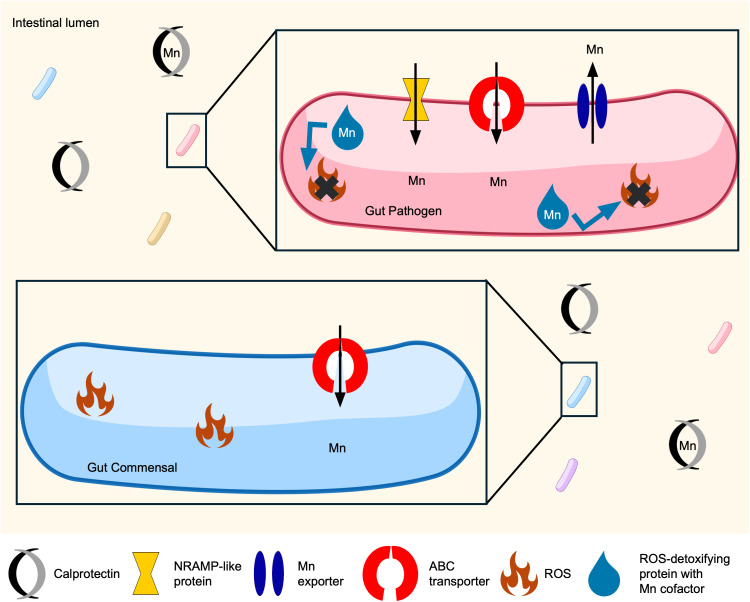
Mn helps pathogens overcome oxidative stress. During infection, Mn is a limited nutrient metal. While gut commensals can import Mn with ABC transporters, pathogens are able to outcompete commensals using NRAMP-like proteins. Gut pathogens can use Mn as a cofactor for ROS-detoxifying proteins, allowing pathogens to persist through host immune-system imposed oxidative stress.

Mn, as both an element and an enzyme cofactor, detoxifies ROS and controls oxidative damage in all domains of life [169–171]. Like other trace metals, excess levels of Mn are toxic due to mismetallation of enzymes and may result in a deficiency of ATP [104,172]. During bacterial infection, vertebrates use two innate immune factors, *n*atural *r*esistance-*a*ssociated *m*acrophage *p*roteins (NRAMP) and calprotectin, to starve pathogens of Mn [173,174]. To obtain sufficient Mn, bacteria have several uptake mechanisms, such as ABC transporters and NRAMP homologs [175–177] (Fig 3). Mn transporters are often regulated by MntR or the Fur-family regulator, Mur [178]. Mn has a biological relationship to Fe such that Fur also represses Mn uptake systems in *Salmonella* Typhimurium and *E. coli* [179,180]. The Mn:Fe ratio within bacterial cells is important for maintaining homeostasis as excess Mn can disrupt heme biosynthesis and subsequently heme-requiring enzymes, such as cytochrome oxidase proteins [181–183]. Furthermore, the oxidative stress sensors PerR in gram-positive bacteria and OxyR in gram-negative bacteria can bind to either Mn or Fe and influence transcription, indicating that both divalent metals are critical to limiting oxidative damage [184,185]. The impact of Mn on the gut microbiota and susceptibility to enteric pathogens is a fruitful area for future research.

### Mn and the gut microbiota

Given that Mn is a critical nutrient metal, it is unsurprising that Mn deficiency has detrimental effects on both vertebrates and their gut microbiota. Mn is important to produce mucopolysaccharides, which make up the GI mucosal barrier, therefore sufficient Mn leads to a robust barrier against pathogens [186]. Indeed, mice have more tolerance to dextran sulfate sodium-induced colitis when fed a Mn supplemented diet relative to a control diet. Moreover, mice with Mn deficiency have increased intestinal permeability, more colonic injury, and higher morbidity from colitis [187]. Further, an increase in intestinal permeability shifts the gut microbiota toward dysbiosis [9]. Accordingly, Mn supplementation in pigs decreases the abundance of pathogenic bacteria, while increasing the abundance of the beneficial genera, *Roseburia* and *Turicibacter* [188]. Overload of Mn can be cytotoxic, resulting in a less diverse gut microbiota with less SCFA being produced, which may lead to more inflammation in the gut [189]. Interestingly, the alterations in gut microbiota composition may be sex-specific. Excess dietary Mn decreased commensal *Bacteroides* spp. and increased Bacillota in male mice while Bacillota abundance decreased in female mice. The sex-specific microbiota differences may be explained by different levels of inflammation in the gut. Indeed, the gut microbiota from Mn-treated female mice has a higher abundance of genes involved with lipopolysaccharide synthesis and assembly, which could lead to more pro-inflammatory cytokines in the GI tract of female mice [190].

### Mn in enteric infections

Although the host sequesters Mn during infection, enteric pathogens have complex systems to acquire Mn. *Salmonella* possesses the NRAMP-like protein MntH and the ABC transporter SitABCD, which allow it to resist oxidative damage and nitrosative stress [191–193]. In fact, these systems enable *Salmonella* to persist even in the presence of calprotectin. *Salmonella* uses Mn as a cofactor for enzymes SodA and KatN, which detoxify ROS and enable the pathogen to outcompete commensal *E. coli* and evade killing by neutrophils [194]. Similarly, KatN is required for enterohemorrhagic *E. coli* pathogenesis by protecting against host-produced ROS [195] (Fig 3). In addition to resisting host antimicrobial defenses, such as oxidative stress, Mn is important for *Salmonella* to maintain central metabolism and energy production [196]. Despite Mn sequestration being an effective immune strategy, Mn supplementation in broiler chicks reduced inflammation and cecal burden of *Salmonella* compared with Mn-deficient broilers [197]. This may be due to the requirement for Mn by the vertebrate immune system or explained by the variance in mammalian and avian immune systems.

The opportunistic pathogen *E. faecalis* relies on Mn acquisition from an ABC transporter and two NRAMP transporters to survive Mn limitation and be fully pathogenic [198]. *E. faecalis* also encodes a cation diffusion facilitator protein, MntE, which exports Mn to alleviate toxicity. MntE is required for *E. faecalis* to colonize the mouse GI tract [199] (Fig 3). The ability of *E. faecalis* to both import and export Mn ensures its survivability in a wide range of Mn concentrations.

### Future directions of Mn research

Mn is important in a variety of human pathogens, such as *Staphylococcus aureus* and *Streptococcus pneumoniae* [200–202]. It is likely that Mn homeostasis influences the pathogenesis of intestinal pathogens, such as *C. jejuni* and *C. difficile*, although this remains to be investigated. There is a profound connection between Mn, gut microbiota health, and the nervous system. In fact, Mn-induced neurotoxicity is modulated by dietary Mn consumption and the resulting gut microbiota [203]. Excess Mn exposure can result in a Parkinson’s-like disease [204]. Rats with Mn in their drinking water have elevated levels of inflammatory cytokines, beta-amyloid, and tau, which are associated with Alzheimer’s disease [189]. Furthermore, Mn-treated rats that received a fecal microbiota transplant, which is usually prescribed to re-establish gut microbial diversity and treat severe CDI, had downregulation of beta-amyloid and tau and reduced activation of the NLRP3 inflammasome [189,205]. These results suggest that a healthy microbiota can protect the nervous system from Mn toxicity. The role of Mn and other trace transition metals in the gut–brain axis should be investigated further to identify mechanisms of protection and evaluate if and how enteric infections impact the nervous system.

## Copper (Cu)

**Fig 4:**
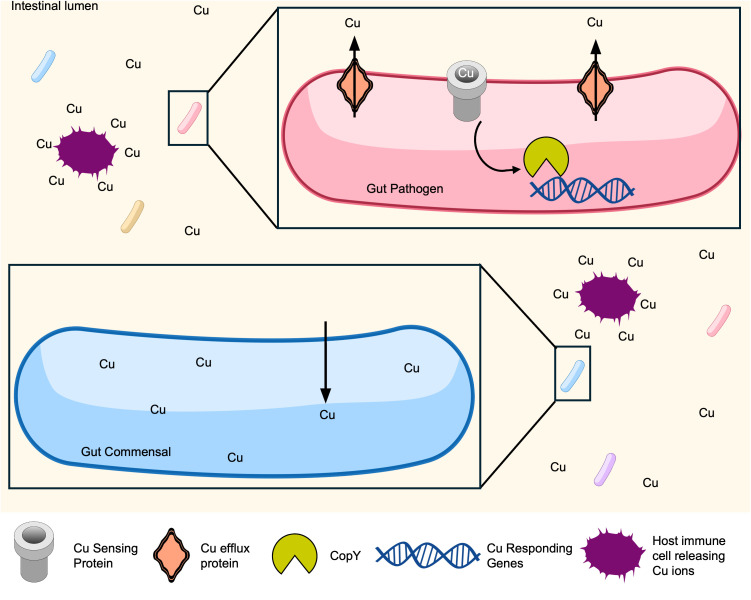
Pathogens overcome Cu toxicity with efflux systems. Contrary to other metals where the host immune strategy is metal limitation, Cu is often present in excess after infection-induced inflammation and can cause toxicity. Pathogens have evolved several Cu sensing mechanisms, such as CopRS, CueRS, and CusRS, which allow transcriptional regulators like CopY to increase expression of Cu efflux systems. Commensals may have less ability to regulate intracellular Cu levels, possibly allowing them to be outcompeted by pathogens.

Cu is a nutrient metal that is essential for central metabolism and a cofactor for enzymes involved in the antioxidant response [206,207]. Cu can cause toxicity through oxidative tissue damage, as Cu can exist as Cu^+^ or Cu^2+^ ions [208]. Contrary to prototypical nutritional immunity which sequesters nutrient metals, vertebrate hosts often increase local Cu concentrations, subjecting pathogens to Cu toxicity. Cu participates in Fenton-like chemistry and can generate cytotoxic levels of ROS [209]. In innate immune cells, Cu accumulates and has an antimicrobial effect against pathogens [19,210] (Fig 4). One method of toxicity is when Cu disrupts iron-sulfur (Fe–S) clusters [211,212]. Fe–S clusters are essential to the structure and function of critical enzymes, such as those involved in amino acid biosynthesis and DNA replication [213,214]. Furthermore, the binding of metals to proteins is dictated by the Irving–Williams series (Mn < Fe < Co < Ni < Cu > Zn), making Cu highly likely to mismetalate proteins [215,216]. To regulate Cu levels, many gram-negative bacteria have the CueRS and CusRS systems, which sense increased Cu levels and regulate gene expression to respond [217–219]. In gram-positive bacteria, there are many sensing and regulating mechanisms including proteins from the *c*opper-*s*ensitive *o*peron *r*epressor (CsoR) superfamily, which sense excess Cu, and transcriptional repressor, CopY [215,220–222] (Fig 4). The role of Cu in the maintenance of the gut microbiota and prevention of gut pathogen infection remains understudied.

### Cu and the gut microbiota

Exposure to excess Cu reduces the prevalence of gut commensals with rats exposed to Cu having dose-dependent decreases in gut microbial diversity. Cu exposure in rats also increases intestinal inflammation and lowers the abundance of beneficial species from Bacillota, *Bacteroides* spp., and *Akkermansia* spp*.* [223,224]. Corroborating this, Chinese brown frogs have less SCFA-producing bacteria after Cu exposure [225]. Similarly, dietary Cu supplementation in pigs reduces the abundance of beneficial *Lactobacilli* in the gut, increases abundance of Enterobacteriaceae, and increases intestinal barrier dysfunction [226]. Altogether, these results indicate that excess Cu in the diet diminishes the gut microbiota and may lead to dysbiosis.

Conversely, Cu sulfate supplementation increases beneficial *Lactobacilli* and reduces *E. coli* in broiler chicks [227]. These differences can be explained by various host species as well as different Cu-containing compounds. Indeed, whether rabbits are supplemented with organic Cu or inorganic Cu compounds has profound impacts on the gut microbiota and the subsequent SCFA levels. Treatment with organic Cu citrate resulted in increased abundance of *Enterrococcus* and *Paeniclostridium* and decreased abundance of *Ruminococcus* compared with inorganic Cu treatment. Organic Cu treatment resulted in less butyrate in rabbit ceca relative to inorganic Cu [228]. Pigs are often over supplemented with Cu because of the known antimicrobial effects, however, it is important to consider that these antimicrobial effects could also harm the native gut microbiota and increase inflammation, harming vertebrate health [229]. Furthermore, *Salmonella* Typhimurium ST34, which is a strain associated with livestock infections, has acquired Cu efflux systems that have increased its resistance to Cu toxicity [230]. This suggests that prolonged use of excess Cu in livestock diets may lead to reduced antimicrobial effects in the future.

### Cu in enteric infections

Multicopper oxidases and some superoxide dismutases are Cu-requiring enzymes that are important for pathogens to reduce oxidative stress and promote survival [231]. CueP is a Cu chaperone that is necessary for activation of superoxide dismutase CII (SodCII) in the periplasm of *Salmonella* Typhimurium and SodCII may contribute to the virulence of *Salmonella* by protecting against antimicrobial peptides [218,232]. Therefore, proper concentrations and distribution of Cu within the bacterial cell may play a role in the fitness and virulence of enteric pathogens [233]. The contribution of Cu to pathogenesis remains an active area of research. More commonly, vertebrate innate immune cells increase Cu uptake during infection and uses Cu overload to prevent severe disease [234]. Cu has an antimicrobial effect on pathogenic *E. coli* when orally supplemented to broiler chicks [235]. As a result, enteric pathogens have Cu efflux mechanisms to persist in these excess Cu conditions. CuiD is a protein in *Salmonella* that extrudes Cu out of the cell and is essential for survival of Cu overload [236]. Some enteric pathogens also have P-type family ATPases that can efflux Cu, such as CopA in *E. coli*, *Salmonella*, and *E. faecalis*, and GolT in *Salmonella* [221,237,238] (Fig 4). CusF is a metallochaperone that delivers Cu to CopA and a transenvelope export system CusCBA in *E. coli* [239,240]. There are likely more unidentified metallochaperones that are important for preventing Cu toxicity in gut pathogens.

Importantly, Cu tends to be reduced to Cu^+^ under anoxic environments, such as the gut, which is more cytotoxic to bacteria than Cu^2+^ [241,242]. When the same Cu concentrations are given to the same bacterial species, the bacteria in anoxic conditions accumulate more intracellular Cu [219,241]. In *E. coli*, excess Cu nonspecifically binds to histidine and cysteine residues, leading to significantly increased protein aggregation *in vitro* [243]. Two Fe–S cluster requiring dehydratases that biosynthesize branched-chain amino acids in *E. coli* are also sensitive to Cu toxicity *in vitro*, leading to growth inhibition when exposed to micromolar doses of Cu [211]. Moreover, Cu may bind to the proteins, IscA and IscU, which are responsible for Fe–S cluster biogenesis, preventing *E. coli* recovery from this form of toxicity [212].

The many pathways of Cu toxicity make Cu an attractive option for developing antimicrobial therapies that treat enteric infections [244]. In fact, supplementation of Cu with the ionophore pyrithione leads to previously resistant *Klebsiella pneumoniae* becoming resensitized to amikacin, an aminoglycoside antimicrobial [245]. This finding suggests Cu could be used in conjunction with current antimicrobials, but for enteric pathogens that thrive on dysbiosis, co-treatment may worsen outcomes. The abundance of *C. difficile* was significantly lower in mice exposed to high Cu levels, but the antimicrobial effects of Cu were counteracted by co-exposure to high levels of tetracycline [246]. The ineffectiveness of cotreatment is likely due to reduced colonization resistance from the native gut microbiota. These findings suggest that Cu alone may be a more specific antimicrobial, which may prevent the gut microbiota from entering dysbiosis during treatment of enteric infections, compared to traditional antibiotics.

### Future directions of Cu research

Future studies looking at Cu homeostasis proteins should use *in vivo* models of infection to demonstrate if there are effects on pathogenicity and determine if Cu-induced protein aggregation is a relevant form of Cu toxicity against enteric pathogens. Another area that warrants future investigation is the intersection of antibiotics and dietary supplementation of Cu. In black soldier fly larvae, Cu supplementation led to a higher abundance of antibiotic resistance genes detected from gut microbiota DNA and enriched the abundance of potential pathogens *E. coli*, *S. enterica*, and *E. faecalis* [247]. A similar trend in mammals is observed where co-exposure to high Cu levels and tetracycline resulted in an increased abundance of antibiotic resistance genes in the gut microbiota of mice compared to mice co-exposed to low Cu levels and tetracycline [246]. Finally, there is potential to engineer gut commensals to excrete excess amounts of Cu, similar to vertebrate innate immune proteins, as a potential method of combatting enteric infections. Future work should tease apart the interactions between antibiotics and Cu and how they influence gut microbiota health and the emergence of gut pathogens.

## Conclusions

The sequestration of nutrient metals by the host and gut microbiota often prevents enteric infection [15,19,51,53,96,162,173,174]. However, enteric and opportunistic pathogens have acquired sophisticated mechanisms to survive the stress of nutrient metal limitation and outcompete the native gut microbiota for sufficient Fe, Zn, and Mn [24,54,56,69,70,75,79,144,192]. Enteric pathogens are efficient at colonizing when the microbiota has entered dysbiosis because nutritional competition is reduced [44,49,103,141,246]. Simultaneously, the metals Fe, Mn, and Cu can result in oxidative stress at high concentrations, so bacteria have intricate metal regulation and efflux systems to maintain homeostasis [23,147,199,215,217,218,221,236,248]. The vertebrate host leverages mismetallation to induce toxicity via excess Zn and Cu [19,105,164,215,234,241]. Therefore, dietary intake of nutrient metals should be considered so that the host is not in metal deficiency or overload, which ensures a diverse gut microbiota and lower susceptibility to enteric pathogen infection [33,35,110,121,133,134,156,226].

Given that vertebrate host proteins like calprotectin and transferrin can sequester several nutrient metals, future studies should focus on cross-metal regulation and acquisition [51,138,163,96,173,249]. Furthermore, some bacterial siderophores, more properly termed metallophores if they bind various metals, such as yersiniabactin, can sequester several nutrient metals [64,72]. Rather than focusing on one metal, simultaneously interrogating multiple trace transition metal levels is more biologically relevant, considering for example that transcriptional factors, such as Fur, regulate the acquisition systems of several metals within bacteria [167,179,180,184,185]. These studies should incorporate longitudinal efforts to ascertain the long-term effects of metal deficiency or overload on the gut microbiota. Many of the current gut microbiota studies that investigate the impact of trace transition metal levels are conducted in animal models, which may not directly translate to human biology [35,114,128,130,186–188,190,223,224,227]. Therefore, future studies should leverage synthetic communities or fecal microbiota transplant from human fecal samples to ensure the biology is applicable to human health [250–252]. These studies should incorporate spatial imaging or sequencing techniques to add an additional layer of resolution and focus on the influence of Mn and Cu, since these metals are understudied [96,97].

More research is necessary to investigate the therapeutic potential of commensal and probiotic bacteria that increase competition for the limited nutrient metals at the site of infection. Increased competition from either natural or engineered bacteria may bolster the effects of nutritional immunity and limit enteric and opportunistic pathogens from colonizing or causing severe disease [15,19,163]. It is also possible that commensals and probiotics can be used to protect the diversity of the gut microbiota or modulate vertebrate trace transition metal levels [14,33,63,72,98,253]. Since Cu induces mismetallation and protein aggregation, Cu and its transporters have the potential to become a new class of antimicrobials [216,241,243,244]. Since Cu is more potently antimicrobial in anoxic conditions, it may be effectively used to reduce enteric infections and modulate the gut microbiota, which are an anaerobic ecosystem [219,241]. Altogether, the opportunity to exploit the competition for nutrient metals and the narrow range of tolerable trace transition metal concentrations to prevent and/or treat enteric bacterial infections warrants further investigation.

## Abbreviations:

ABC: ATP-binding cassette
CDI: *Clostridioides difficile* infection
CsoR: copper-sensitive operon repressor
Dps: DNA-binding protein from starved cells
Fur: ferric uptake regulator
GI: gastrointestinal
NRAMP: natural resistance-associated macrophage proteins
ROS: reactive oxygen species
SCFA: short chain fatty acid
SodCII: superoxide dismutase CII
Xus: xenosiderophore utilization system
Zur: Zn uptake regulator
